# First report of the molecular detection of human pathogen *Rickettsia raoultii* in ticks from the Republic of Korea

**DOI:** 10.1186/s13071-021-04695-5

**Published:** 2021-04-07

**Authors:** Misbah Tariq, Jun-Won Seo, Da Young Kim, Merlin Jayalal Lawrence Panchali, Na Ra Yun, You Mi Lee, Choon-Mee Kim, Dong-Min Kim

**Affiliations:** 1 Department of Internal Medicine, College of Medicine, Chosun University, Gwangju, Republic of Korea; 2 Department of Premedical Science, College of Medicine, Chosun University, Gwangju, Republic of Korea

**Keywords:** *Rickettsia raoultii*, Ticks, *Haemaphysalis longicornis*, Spotted fever group

## Abstract

**Background:**

Rickettsial diseases associated with the spotted fever group constitute a growing number of newly identified *Rickettsia* pathogens and their tick vectors in various parts of the world. At least 15 distinct tick species belonging to six genera have shown the presence of *Rickettsia raoultii*. Herein, we report the detection of *R. raoultii* in ticks from the Republic of Korea (ROK).

**Methods:**

Thirty-five ticks were collected from 29 patients with tick bites in Gwangju Metropolitan City, Jeollanam Province, ROK. The ticks were identified using molecular, morphological, and taxonomic characteristics. All samples were screened for presence of *Rickettsia* species using nested polymerase chain reactions of their outer membrane protein (*ompA*) and citrate synthase (*gltA*) genes. The amplified products were sequenced for subsequent phylogenetic analyses.

**Results:**

Sequencing data showed the DNA sequences of *R. raoultii* in three *Haemaphysalis longicornis* ticks. All three tick samples were 99.4–100% similar to previously reported partial sequences of *ompA* of *R. raoultii* strains CP019435 and MF002523, which formed a single clade with the reference strains.

**Conclusions:**

We provide the first description and molecular identification of *R. raoultii* detected in *H. longicornis* ticks in the ROK. This observation extends the geographical distribution of *R. raoultii*. Screening of human samples for this pathogen will provide information about the prevalence of rickettsial infections in this region. 
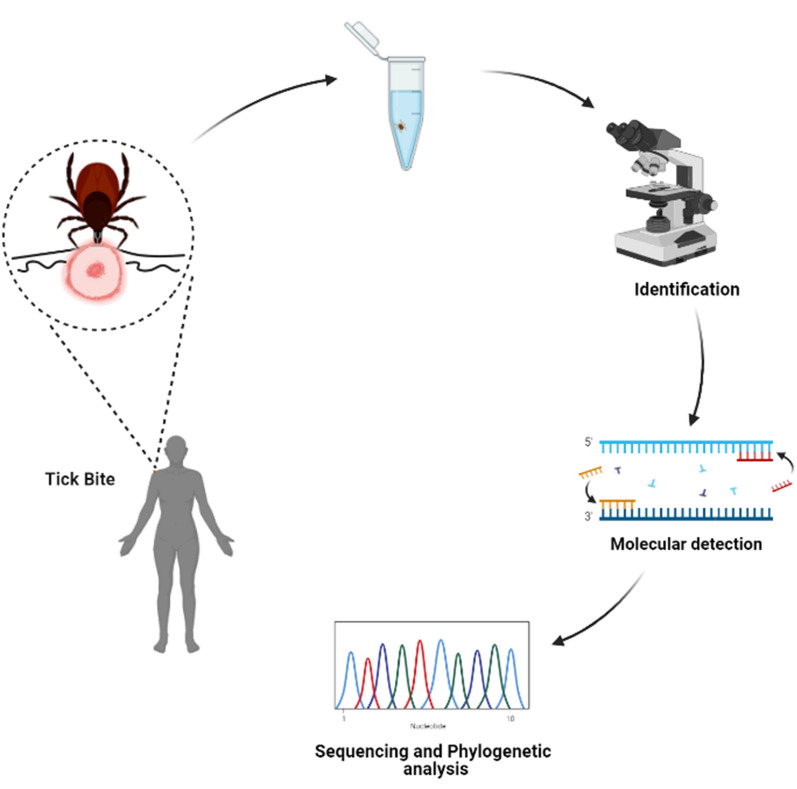

## Background

Tick-borne diseases are a growing medical concern worldwide. Ticks are considered the main reservoirs and vectors of *Rickettsia*, an obligate intracellular bacteria, responsible for the transmission of rickettsial diseases to humans. The rickettsioses represent some of the oldest and most recently recognized infectious diseases [1]. The causative agents belong to the genus *Rickettsia* and are presently classified into four groups: the spotted fever group (SFG), typhus group, *Rickettsia bellii* group, and *Rickettsia canadensis* group [2]. SFG rickettsioses constitute newly identified *Rickettsia* species around the world. In the past few decades, numerous species of tick-borne rickettsiae, previously thought to be non-pathogenic, were recognized as human pathogens [2].

In 1999, three novel rickettsial genotypes, RpA4, DnS14, and DnS28, were observed in ticks from Russia [3]. Using genotypic and phenotypic analyses, these bacteria were recognized as novel species of SFG rickettsiae, and in 2008, the species was designated *Rickettsia raoultii* [4]. The major clinical manifestations of *R. raoultii* infections include scalp eschar and neck lymphadenopathy. Initially, these were termed *Dermacentor*-borne necrosis erythema and lymphadenopathy or tick-borne lymphadenopathy [2]. *R. raoultii* has been identified in many Asian and European countries [5–8]. In 1999, *Dermacentor nuttalli* and *Rhipicephalus pumilio* ticks collected in the southern parts of the former Soviet Union were shown to harbor these bacteria [3]; thereafter, other species of *Dermacentor* ticks (i.e., *D. reticulatus*, *D. marginatus, D. silvarum*, *and D. niveus*) from various parts of the former Soviet Union, as well as from France, Spain, and Germany, were also shown to carry these bacteria [5, 9]. Subsequently, *R. raoultii* was detected in other hard ticks too, such as *Haemaphysalis*, *Rhipicephalus*, *Hyalomma*, and *Ambylomma*, which are observed predominantly in Europe and Asia [10]. The aim of this study was to determine the presence of *R. raoultii* in ticks and to assess the circulation of this pathogen in tick populations in the Republic of Korea (ROK). We found *R. raoultii* in *Haemaphysalis longicornis* ticks. To the best of our knowledge, this is the first report providing molecular evidence of *R. raoultii* in ticks from the ROK.

## Methods

### Tick sampling and classification

In 2018, 35 ticks were collected from 29 patients with a history of tick bites in Gwangju Metropolitan City, Jeollanam Province, ROK. Ticks were identified on the basis of their molecular, morphological, and standard taxonomic characteristics. Briefly, the ticks were first decontaminated using 70% ethanol, rinsed twice using sterile phosphate-buffered saline (PBS), and dried on sterile filter paper. Each sample was then placed in a hard-tissue-grinding MK28 tube (Bertin Technology, Rockville, MD, USA) containing 800 µl PBS and 1× PC/SM (i.e., penicillin and streptomycin). Subsequently, ticks were ground using a FastPrep-24 Classic instrument (MP Biomedicals, Solon, OH, USA) and were stored at − 80 °C until DNA extraction.

### DNA extraction

Total genomic DNA was extracted from 150 µl of the tick homogenate and from 300 µl of whole blood of the respective patients using a QIAamp Tissue & Blood Mini Kit (Qiagen, Hilden, Germany) according to the manufacturer’s instructions; the DNA was eluted in volumes of 50 µl and 100 µl, respectively. The samples were stored at − 20 °C until polymerase chain reaction (PCR) amplification.

### PCR amplification

For molecular identification, tick genomic DNA was subjected to PCR amplification of a fragment of the mitochondrial 16S rRNA gene [11]. To assess the presence of *Rickettsia* species in ticks and patients, genomic DNA samples were subjected to a nested PCR targeting the outer membrane protein A (*ompA*) and citrate synthase *(gltA)* genes [12, 13]. PCR primers and the respective product sizes are shown in Table 1. The reactions were carried out in a total volume of 20 µl, comprising 16 µl distilled water, 1 µl of each primer (10 pmol/µl), and 2 µl genomic DNA template using AccuPower PCR PreMix (Bioneer, Daejeon, ROK). The PCR analysis was performed using an AB thermal cycler (Applied Biosystems, Foster City, CA, USA). A positive control with *R. conorii* DNA and a negative control with distilled water instead of template DNA were included in each set of PCR. The amplified products were analyzed by electrophoresis using a 1.2% agarose gel containing ethidium bromide and then visualized by using an ultra-violet transilluminator system (FAS-III, Toyobo, Osaka, Japan). A 100-bp ladder (Bioneer Corp, Korea) was used as a molecular weight marker.

**Table 1:** Oligonucleotide primers that were used to perform PCR in this study to detect the molecular targets in *Rickettsia* species

Target	Primer	Nucleotide sequence (5′–3′)	Fragment length	Reference
*ompA*	RR190.70F	ATGGCGAATATTTCTCCAAAAA	634 bp (first)	[12]
	RR190.701R	GTTCCGTTAATGGCAGCATCT		
	RR190.70F	ATGGCGAATATTTCTCCAAAAA	535 bp (nested)	
	RR190.602R	AGTGCAGCATTCGCTCCCCCT		
*gltA*	GLTA1F	GACGGTGATAAAGGAATCTTG	1022 bp (first)	[13]
	GLTA1R	CATTTCTTTCCATTGTGCCATC		
	GLTA2F	CTACGAACTTACCGCTATTAG	446 bp (nested)	
	GLTA2R	GACCAAAACCCATTAACCTAAAC		

### Phylogenetic analysis

The PCR products were purified using a QIAquick PCR purification kit (Qiagen) and were sequenced in both directions by a commercial service provider (Solgent Inc, Daejeon, Korea). To analyze the percentage of similarity, the resulting sequences were correlated for identity with sequences from GenBank using the Basic Local Alignment Search Tool (BLAST) program. The neighbor-joining method was employed to produce a phylogenetic tree with the ClustalW algorithm of the MegAlign program (DNASTAR, Madison, WI, USA). Bootstrap analysis was performed to test the stability of the phylogenetic tree acquired through the neighbor-joining method.

## Results

Molecular, morphological, and taxonomic characteristics revealed that 4 of the 35 ticks were *Ixodes nipponensis*, 14 were *Amblyomma testudinarium*, and 17 were *H. longicornis* (Table 2). PCR tests to amplify the *ompA* and *gltA* gene fragments for identification of SFG rickettsial disease agents were conducted on all 35 ticks. Sequencing data of the amplified *ompA* gene fragment revealed one distinct *Rickettsia* species in three *H. longicornis* ticks, which was identified as *R. raoultii.* Morphological and taxonomic characteristic showed that these ticks were adult females. The PCR targeting the *gltA* gene did not reveal any distinct *Rickettsia* species.

**Table 2:** Tick species observed in this study based on the stages of development and sex, as per their collection from patients with tick bites

Tick species	*Haemaphysalis longicornis*	*Amblyomma testudinarium*	*Ixodes nipponensis*
Developmental stage			
Adult female	13	–	4
Adult male	–	5	–
Nymph	1	9	–
Larva	3	–	–
Total no.	17	14	4
	35		

**Fig. 1:**
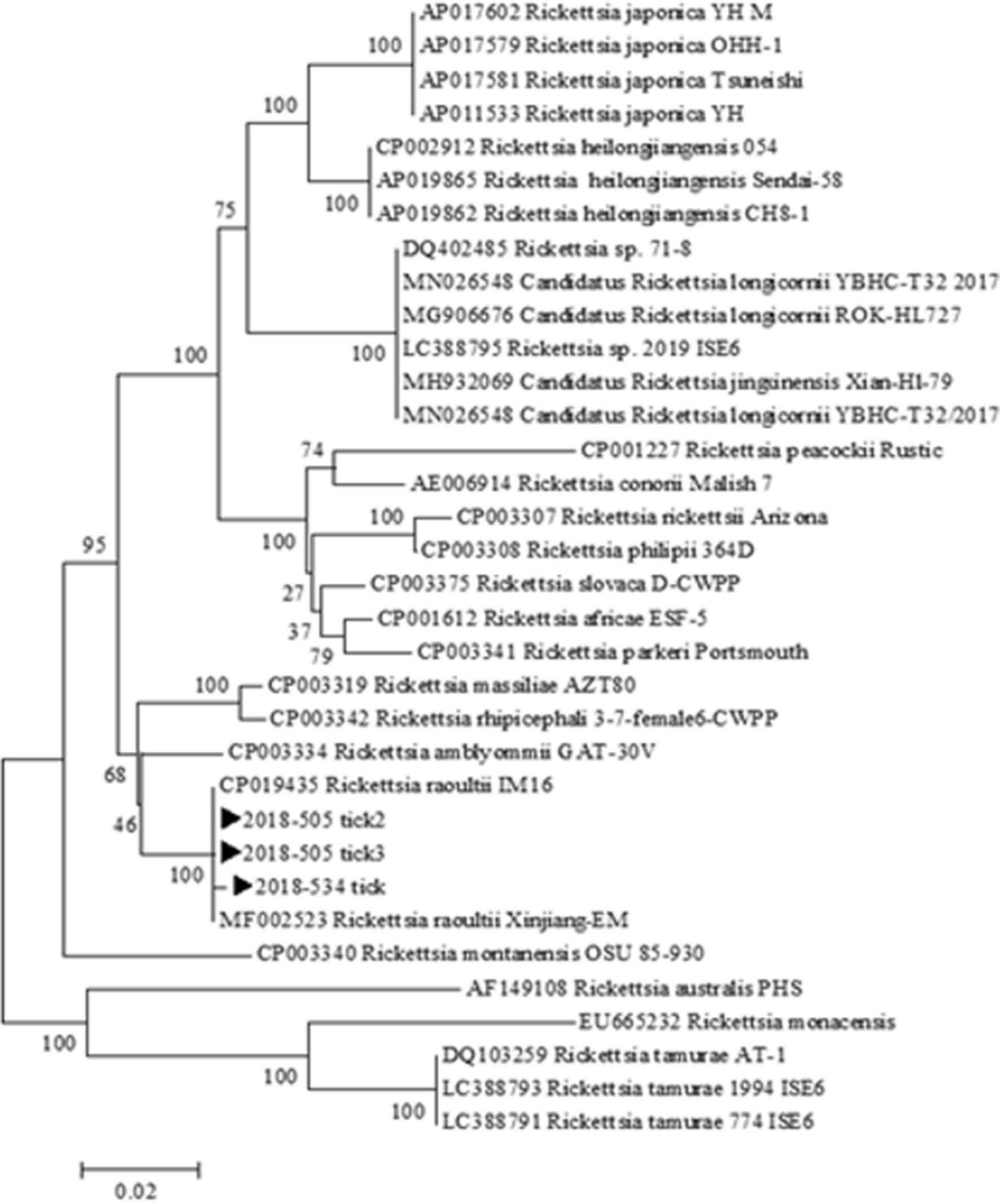
Phylogenetic analysis of *Rickettsia* based on a partial (487-bp) *ompA* gene. Concatenated sequences are *Rickettsia raoultii* from *Haemaphysalis longicornis* ticks acquired in this study (►) and sequences of *Rickettsia* downloaded from the GenBank database. The phylogenetic tree was produced using a neighbor-joining algorithm (NJ-1000 bootstrap trials)

Even though the three *R. raoultii*-positive ticks were collected from patients, blood samples from the respective three patients did not show *R. raoultii* infection, as assessed by PCR, nor did the patients show any symptoms suggesting infection by this pathogen. *R. raoultii ompA* sequences of all three tick samples were 99.4%–100% homologous to previously reported partial sequences of *ompA* from *R. raoultii* IM-16 strains isolated from CP019435 and MF002523. Using phylogenetic analyses, a neighbor-joining tree of *Rickettsia* species indicated that isolates of the current study belonged to a single clade with *R. raoultii* reference strains (Fig. 1). The bootstrap analyses statistically supported the main clustered sequence.

## Discussion

Currently, 14 distinct tick species from 6 genera including *Dermacentor* (*D. nuttalli, D. reticulatus*, *D. silvarum*, and *D. marginatus*), *Ambylomma* (*A. helvolum*), *Haemaphysalis* (*H. concinna*, *H. japonica*, *H. erinacei*, and *H. longicornis*), *Hyalomma* (*Hy. asiaticum* and *Hy. lusitanicum*), *Ixodes* (*I. persulcatus* and *I. ricinus*), and *Rhipicephalus* (*Rh. pumilio* and *Rh. turanicus*) have shown the presence of *R. raoultii* [14, 15]. This bacterium was also found in *Melophagus ovinus*, a louse fly or sheep ked [16]. *Dermacentor* ticks are considered the main hosts and natural reservoirs of *R. raoultii* all over Europe and in a few countries in Asia, including China and Mongolia [17].

In the ROK, the first evidence of SFG rickettsiae in ticks was reported in 2003, followed by the first case of SFG rickettsiosis (Japanese spotted fever) in a patient in 2005 [18, 19]. Over a period of 16 years, various species of SFG rickettsiae have been identified in ticks (*R. japonica*, *R. monacensis*, and *R. rickettsii)* and humans (*R. japonica* and *R. monacensis*) in the ROK [18–24]. Thus far, *R. raoultii* had not been identified in this region, and the present study reports its detection for the first time. Previously, only one study from China indicated the presence of *R. raoultii* in *H. longicornis* ticks [15]. Different strains of *R. raoultii* including Marne, 8/9 Karaganda, Khabarovask^T^, Shayman, and IM 16 have been documented in Europe, Russia, and China [5, 10]. The phylogenetic tree produced in the current study showed that the positive samples formed a distinct clade at a high (100) bootstrap value with *R. raoultii* strain IM 16 from China. DNA sequences of *R. japonica*, *R. monacensis*, and *R. rickettsii* have been identified in *H. longicornis* from ROK [10, 18]. According to a recent study, *H. longicornis* is the most prevalent tick species in the ROK (88.9%), showing a nationwide distribution [24]. Despite its identification in multiple tick species, reports on human infections with *R. raoultii* are still lacking. Infections of patients with *R. raoultii* have been reported in Europe, the Far East of Russia, and a few cases in China [25]. Another study from China [17] identified *R. raoultii* DNA in clinical samples, apart from positive serological reports in patients from other countries. Based on these findings, *R. raoultii* is considered a human pathogen [26]. The observations of the current study indicate the presence of *R. raoultii* in ticks in the ROK, which warrants further research.

## Conclusions

Our results provide evidence for the first instance to our knowledge of the identification of *R. raoultii* in ticks from the ROK. Detection of *Rickettsia* species in *H. longicornis* ticks suggests that these ticks may be a vector of this pathogen in the ROK. This observation broadens our knowledge of the geographical distribution of *R. raoultii*. Even though no human clinical infection was observed, the high pathogenicity of this bacterium is a major concern for public health in this region. Further extensive research in a broader range of ticks and surveillance programs in this regard are therefore required.

## Data Availability

The datasets analyzed during the current study are available in the GenBank-database with the previously reported accession numbers; CP019435 and MF002523.

## Abbreviations

SFG: Spotted fever group
ROK: Republic of Korea
*ompA*: Outer membrane protein A
PBS: Phosphate-buffered saline
PCR: Polymerase chain reaction
